# Meiotic Models to Explain Classical Linkage, Pseudolinkage, and Chromosomal Pairing in Tetraploid Derivative Salmonid Genomes: II. Wright is Still Right

**DOI:** 10.1093/jhered/esv056

**Published:** 2015-08-29

**Authors:** Bernie May, Mary E. Delany

**Affiliations:** From the Department of Animal Science, University of California at Davis, Davis, CA 95616.

The purpose of the article by Allendorf et al. (2015) in the words of the authors is *“(1) to synthesize what is known about the transmission genetics of salmonid fishes and (2) to consider how ignoring these patterns could lead to erroneous conclusions about their genomic organization and population genetics.*” The first section of the article is designed to bring the reader up to speed on what is currently known about transmission genetics in salmonid fishes. The glossary of terms is an important addition to this effort. Allendorf et al. (2015), explain very well the issues surrounding the practice and significance of ignoring loci in the telomeric regions of homeologous arms and this section will be of interest to all geneticists who work on polyploid and polyploid derivative organisms. However, the chromosomal pairing models presented are misleading, as they do not incorporate the available chromosomal evidence. Further their explanations of how their models differ from Wright et al. (1983) are unsupported and confusing given the models presented. Herein we elaborate on the chromosomal pairing models of Wright et al. (1983) based on cytogenetic evidence to improve understanding of the intriguing complex chromosomal pairing and segregation patterns observed in salmonid male meioses.

The chromosomal pattern for residual tetrasomy (Figure 1; Allendorf et al. 2015) derives from earlier work, starting with an original proposal by Allendorf and Thorgaard (1984, see Figure 4 therein). In the 1984 article, they showed a model of 4 acrocentrics in a multivalent pairing configuration. The original model had only one crossover between 2 of the homeologous arms of the acrocentrics. They proposed this as a general model with no specificity regarding chromosomal behavior. An update by Allendorf and Danzmann (1997) added 2 crossovers in the disomic regions of the homologous chromosome arms close to centromeres, keeping the acrocentric model and assuming 3 crossover events among these 4 arms. This was also proposed to be a general model, but in fact is even more complex and ignored the available chromosomal evidence (Davisson et al. 1973; Lee and Wright 1981). Allendorf and Danzmann (1997) state “*we have no direct chromosomal evidence for this model in salmonids*”.

**Figure 1.**
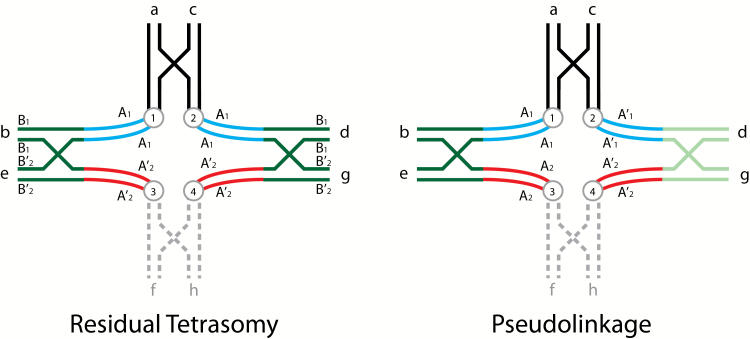
Diagrammatic illustrations of tetravalent pairing of homologous regions of homeologous chromosome arms illustrating both residual tetrasomy and pseudolinkage in salmonid males (adapted from Wright et al. 1983). Residual tetrasomy is illustrated on the left side with chromosomal arms labeled in lower case letters and color coded, centromeres appear as circles with letters, 2 loci are shown, where the A locus is proximal to the centromere (centromeric) and the B locus is distal (telomeric), and a single crossover event is shown in the telomeric region of each pair of arms. Arms f and h are shown in broken grey lines to allow the reader to view this tetravalent as 2 metacentrics (ab and cd) and either 2 acrocentrics (e and g) or another set of metacentrics (ef and gh). The a and c arms, the b and d arms, and the e and g are homologous (as would be f and h). Arms b and d are homeologous with respect to e and g, that is, they were ancestrally homologous, but have diverged. Using arm b as an example, it is composed of 2 general regions where the dark green telomeric region is homologous to not only the telomeric region of d, but it is also still homologous with the telomeric regions of e and g (also dark green), and can pair homologously in the telomeric region (under the male meiotic environment) with either e or g. The centromeric area of b (blue) is homologous with the centromeric region of d and they can pair as bivalents. The centromeric area of b is homeologous with e and g (red) and cannot pair in these regions with e and g. The homologous telomeric regions (dark green) are residually tetrasomic and the centromeric regions (blue and red) are disomic, that is, the A loci have diverged and are respectively disomic and the B loci can exhibit disomic or tetrasomic pairing and are isoloci (sharing the same alleles). We show these centromeric areas separated from each other in this figure. When these arms separate via alternate disjunction centromeres 1 and 4 will go to one meiotic pole and centromeres 2 and 3 will go to the other pole. If this is one set of metacentrics and one set of acrocentrics the chiasma will terminalize forming a long rod, or a ring if there are 2 sets of metacentrics involved. In some meioses this tetravalent will form and in other meioses only the 2 respective bivalents will form. The ratio of bivalent versus tetravalent formation will give the intermediate results for the B locus shown for males in Table 1 of Allendorf et al. (2015). The 2 A loci will show random assortment. The pseudolinkage figure is similar to that shown for residual tetrasomy, except that the A locus alleles are labeled differently where the A comes from one parent and the A’ from the other parent, the B locus is dropped, and homologous distal regions of the homeologous arms are shaded differently. For discussion purposes, we will consider this figure to represent meiotic pairing in a male splake (hybrid between lake and brook/speckled trout). Note: the parental genomes could have come from divergent populations of the same species. Arms c, d, g(gh) come from the lake trout parent and arms a, b, e(ef) from the brook trout parent. In this case a and c, both shown in black will pair since they have no homeologues, (similarly f and h), even though there may be substantial differentiation. The centromeric regions of b and d are both shown in blue and likewise the centromeric regions of e and g are still both shown in red, even they have clearly diverged from one another, in order to reduce the number of colors. The telomeric region of arm b (dark green) will preferentially pair with e (dark green), while arm d (light green) will pair with g (light green) because of their greater homology. This preferential pairing is what makes pseudolinkage a specialized case of residual tetrasomy. Alternate disjunction (centromeres 1 and 4 going to one pole and centromeres 2 and 3 going to the other) will lead to the formation of only non-parental gametes A’_1_ and A_2_ and A_1_ and A’_2_ every time this tetravalent forms since there is no crossing over between the A locus and the centromere. Parental and non-parental gametes of the 2 unlinked A loci will be produced in equal frequencies whenever bivalents of these chromosome are formed. Again, the ratio of formation of bivalent versus tetravalent pairing will determine the extent of the pseudolinkage of the 2 disomic A loci. We (and Allendorf et al. 2015) have limited allelic variation within these models to illustrate the phenomena without introducing the complexity of within locus variability. The reader is encouraged to add allelic variation at each locus within and between each parental chromosome to see the gametic possibilities.

In Figure 1 of Allendorf et al. (2015), a set of metacentric chromosomes replaces one of the sets of acrocentrics and one of the crossovers is moved from one side of the centromere to the opposite arm of the newly added metacentric. The essence of this model is that at first the homologous chromosomes pair completely and then the homeologous arms pair, introducing an additional crossover event in one of the arms already involved in a crossover. While the authors cite evidence from other organisms for secondary pairing of chromosomes already paired, it is not the most parsimonious explanation for residual tetrasomy in salmonids. The caveat proposed in support of their model in the authors’ words is “*Figure 1 shows one of several possible chiasmata formations where this model would support equational division*.”, but this caveat does not lend any more support for this model. What are some of these other possible chiasmata, other than that proposed by the Wright model? It is unclear how this configuration could unfold and give the long multivalent rods observed during Meiosis I.

In Figure 3, Allendorf et al. (2015) have drawn up a new model to explain pseudolinkage. Surprisingly, their model for pseudolinkage, unlike in Figure 1 for residual tetrasomy, does not involve 4 chromosome arms pairing and multiple crossovers in the same arm. This proposal would suggest that residual tetrasomy and pseudolinkage are 2 different phenomena and not that pseudolinkage is a specialized case of residual tetrasomy. It is interesting that they show 2 metacentrics and 2 accrocentrics for residual tetrasomy and 4 metacentrics for pseudolinkage. Further, Figure 3, although technically correct in pairing and crossover events, is displayed in a fashion making it largely impossible to understand chromosomal segregation. Note the extra-large crossover event and the inversion of the grey arms into proximity with the black arms that are clearly neither homologous nor homeologous.

In the late 1970s and early 1980s the late Dr James E. Wright, Jr., who had been studying salmonid meiotic chromosome pairing in males and females simultaneously with allozyme segregation patterns for over 10 years, assembled a team of graduate students and technicians who worked together on the unusual patterns of “multivalent” pairing in male meioses and allozyme segregation patterns. These 2 lines of research and the resultant data were integrated to formulate robust models that fit both chromosomal pairing patterns and allozyme segregation patterns that could explain both residual tetrasomy (May et al. 1979) and pseudolinkage (Morrison 1970; Davisson et al. 1973), showing pseudolinkage to be *simply* a specialized case of residual tetrasomy. The authors of this article were members of that team.

Allendorf et al. (2015) define homeologous as “*partially homologous chromosomes indicating original ancestral homology*”. While we agree with the use of this term to refer to chromosomes and more importantly to specific sets of chromosome arms, the term “homeologous” should not be used as a modifier in regard to “homeologous pairing”, “homeologous crossovers”, “homeologous recombination”, or “homeologous exchanges”. We believe all of these uses have led to some of the confusion in understanding residual tetrasomy and pseudolinkage and are more correctly termed as “homologous” because the terminal ends of homeologous arms where pairing occurs are truly homologous. There is no pairing in the homeologous regions. Hence the reason there are residually tetrasomic isoloci in these telomeric regions and disomic loci proximal to the centromere.

Below we re-present the models of Wright et al. (1983) to help understand residual tetrasomy and pseudolinkage. In Figure 1 of this article, we show models for both phenomena, and their relationship to one another. We maintain the Allendorf et al. (2015) designation of loci; the A loci being close to the centromere and disomic and the B loci being telomeric and isoloci (residually tetrasomic). In Figure 2, we show models of how the pachytene pairing (Figure 1) unfolds to be seen in metaphase I chromosomal spreads (Figure 3). We include the unfolding of bivalents as well. In Figure 3, we show chromosomes generated from male meiotic cells for a rainbow trout and a brown trout to present the reader with images of real chromosomes engaged in both bivalent and multivalent pairing.

**Figure 2.**
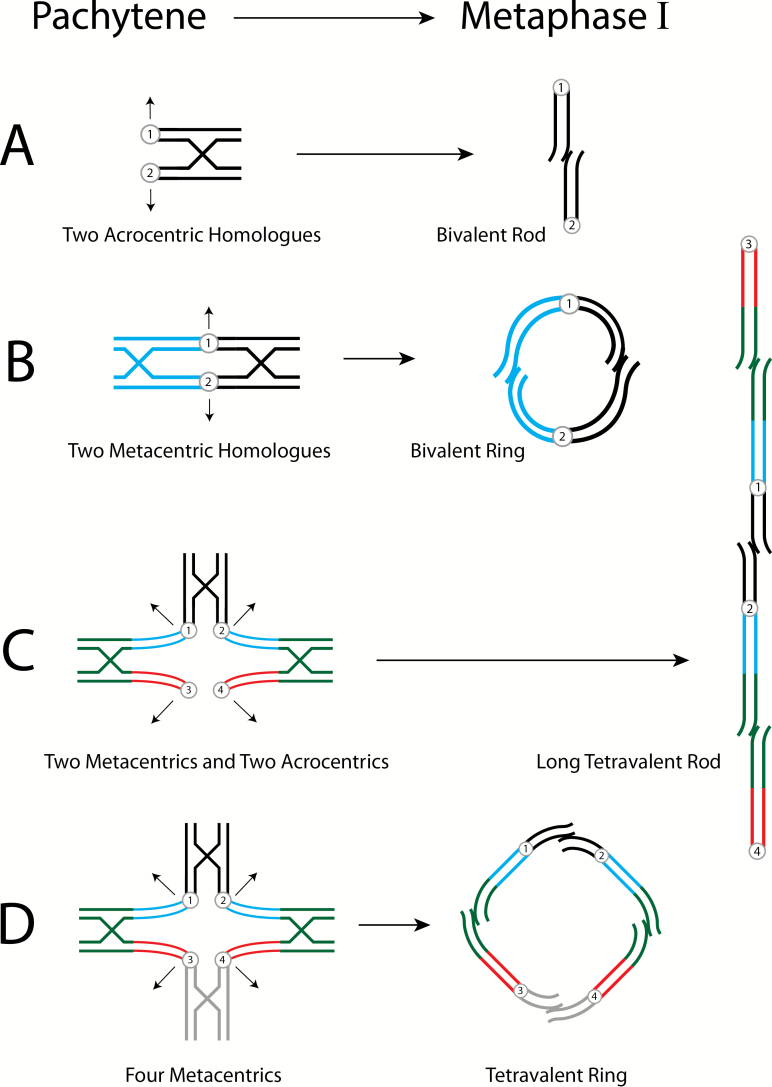
Diagrammatic interpretation of male salmonid pairing configurations, Pachytene to Metaphase of Meiosis I. Sister chromatids are adjacent, circles with numbers denote centromeres. Recombination and chiasmata terminalization indicated by cross structures; arrows indicate direction of the chromosomes ‘opening’ to opposite poles. (**A**) Bivalent pairing of 2 acrocentrics, disomic homologues (black) opens to a small rod. (**B**) Bivalent pairing of 2 metacentrics, disomic homologues (blue and black) opens to a small ring. (**C**) Tetravalent pairing involving 2 metacentrics (homologues) and 2 acrocentrics (homologues) which opens to a long mulitvalent rod. The homologous telomeric ends of the homeologous metacentrics and acrocentrics pair, while the disomic centromeric regions do not. See Figure 1 for a more detailed description of the tetravalent. It is important to remember in all of these figures that they are 2-dimensional compressions of 3-dimensional phenomena. Centromeres 1 and 4 which are moving to one meiotic pole can be considered to be above the page and centromeres 2 and 3 which are moving to the opposite pole can be considered to be below the page. (**D**) Tetravalent pairing of 4 metacentrics that opens to a large tetravalent ring. This is similar to 2C except the acrocentric pair is replaced by a pair of metacentrics.

**Figure 3.**
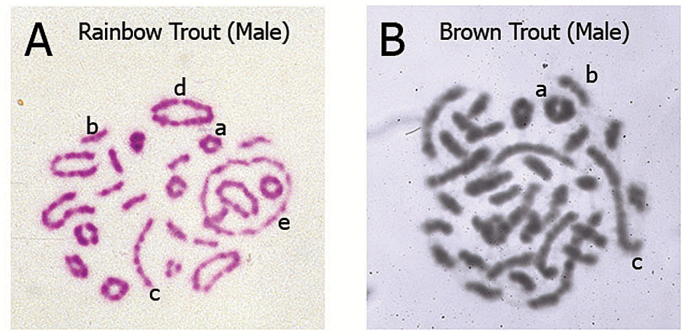
Meiosis I metaphase stage chromosome pairing configurations of male salmonids with different karyotype architecture: bivalent and multivalent pairing results from homologous and homeologous ancestral relationships. Explanation of the configurations is provided in the context of major events of Meiosis I. Chromosome regions that synapse (pair) and recombine (undergo crossing-over) during Prophase I (pachytene) will move to align and orient on the Metaphase I equitorial plate via the action of centriole-derived spindle fibers which attach to the centromeres and create tension on the chromosomes. The polymerization/depolymerization action of the spindle fibers results in the pulling of alternating chromosomes toward the opposite poles of the dividing cell. Thus, a cross structure in Prophase I involving 4 metacentrics becomes a large ring of 4 chromosomes (tetravalent) as the chiasmata terminalize (move to the ends of the chromosome), see Figure 2D. Ultimately the maternal and paternal chromosomes segregate (assort) independently and accurately during the reductional division of Meiosis I (one diploid 2*n* cell divides to produce 2 haploid 1n cells). The types of pairing configurations observed relates directly to the architecture of the karyotype. That is, the number of metacentrics and acrocentrics found in the karyotype which varies among salmonid species due to the variable number of Robertsonian centric fusions which occurred during species radiation following the ancestral tetraploid event. (**A**) Representative example of rainbow trout (*Oncorhynchus mykiss*) bivalent and multivalent pairing configurations (Giemsa stained, color image). Typically, rainbow trout show several large multivalent rings (4, 6, or 8 metacentrics), a lesser number of long multivalent rods (2 metacentrics/2 acrocentrics) and bivalent pairing (small rings of 2 metacentrics and small rods of 2 acrocentrics). This image is of a cell from a fish from a Benner Springs Hatchery (PA) population, 2*n* = 64 (40 metacentrics, 24 acrocentrics; 104 arms). (**B**) Representative example of brown trout (*Salmo trutta*) bivalent and multivalent pairing configurations (Giemsa stained, black/white image). Typically, brown trout show several long multivalent rods (2 metacentrics/2 acrocentrics) and bivalent pairing (a low number of small rings of 2 metacentrics and numerous small rods of 2 acrocentrics). This image is of a cell from a fish from a Benner Springs Hatchery (PA) population, 2*n* = 80 (20 metacentrics, 60 acrocentrics; 100 arms). Representative configurations indicated by the lowercase letters in A and B: (a) small ring bivalent of 2 metacentrics pairing (b) small rod bivalent of 2 acrocentrics pairing (c) long rod multivalent (tetravalent) of an acrocentric–metacentric–metacentric–acrocentric pairing, common in species with high numbers of acrocentrics (d) large ring multivalent (tetravalent) of 4 metacentrics pairing, common in species with high numbers of metacentrics (e) large ring multivalent (hexavalent) of 6 metacentrics pairing, common in species with high numbers of metacentrics. The chromosome preparations shown were created from cells harvested from male salmonids in 1980 (MED) in the cytogenetics laboratory of the late James E. Wright, Jr., at The Pennsylvania State University, State College, PA and supported by an NSF grant to JEW in 1979.

Figure 1 presents models for the pairing of chromosomes for residual tetrasomy and pseudolinkage, illustrating both the pairing of a set of 2 metacentrics with a set of 2 acrocentrics or the pairing of 2 sets of metacentrics (see the dashed gray arms). For residual tetrasomy, we show the homologous pairing of homeologous arm b with e and d with g. In fact the pairing has an equal likelihood of being b with g and d with e. We see no apparent association (see pseudolinkage below) between the 2 disomic loci A_1_ and A_2_ from gamete segregation because the telomeric regions of the homeologous arms homologously pair randomly. At the same time, ab and cd can pair bivalently and e (or ef) and g (or gh) can pair bivalently. The ratio of bivalent versus tetravalent pairing gives the intermediate segregation results for males seen in Table 1 of Allendorf et al. (2015). Centromeres (1 and 4) for ab and g(gh) move toward 1 meiotic pole and the centromeres (2 and 3) for cd and e(ef) move toward the other, resulting in 2-dimensional chromosome images of a long rod (2 metacentrics and 2 acrocentrics) or a large ring (4 metacentrics, ring of four). The Wright model supports 2 crossover events in the telomeric regions, while the Allendorf et al. model gives only one in their Figure 1 and therefore only half the recombinant events. (Note: Their pseudolinkage model gives the same 2 crossover events as the Wright model). Higher order multivalents can form if 1 pair of metacentrics is homeologous in each of its arms with 2 other sets of chromosomes. Finally, we do not know exactly where the crossover events occur, though we know they do not occur in the centromeric regions (A loci), otherwise these centromeric loci would not have diverged. We show these centromeric regions of the homeologous arms as being separated in space to illustrate this lack of pairing. Lubieniecki et al. (2010) provide data showing “…*there is a strong tendency for recombinations to occur at the ends of linkage groups in the male*.” and “…*recombinations in females are more broadly distributed along the chromosomes*.” This “tendency” in males is undoubtedly due to the ratio of tetravalent pairing to bivalent pairing (bivalent pairing allowing more random crossovers along the arm).

In Figure 2, we adapt the depiction of chromosomal behavior from Lee and Wright (1981, their Figure 3) of pairing configurations moving from Pachytene (our Figure 1) to resultant Metaphase I for a bivalent rod, bivalent ring, tetravalent rod, and tetravalent ring (seen in our Figure 3). Arrows depict the directional movement of the numbered centromeres towards poles allowing for the unfolding of the pairing and crossing over, and the terminalization of the chiasma. Note that all of our diagrams and meiotic figures are 2-dimensional compressions of the actual 3-dimensional chromosome configurations. For example, in Figure 2C centromeres 1 and 4 might be above the page and centromeres 2 and 3 below the page and in our depictions of terminalization the small telomeric ends would not likely be in the same plane.

In Figure 3A, we show actual meiotic chromosomes (Metaphase I) from a male rainbow trout. In this figure, one can observe bivalent pairing of metacentrics (a), bivalent pairing of acrocentrics (b), tetravalent long rods composed of 2 metacentrics and 2 acrocentric homologously pairing in the telomeric regions of their homeologous arms (c), tetravalent rings composed of 4 metacentrics homologously pairing in the telomeric regions of their homeologous arms (d, “ring of four”), and a hexavalent large ring composed of 6 metacentrics homologously pairing in the telomeric regions of their homeologous arms where 2 of the metacentrics share 1 homeologous arm with one of set of metacentrics and the other arm shared with the third set of metacentrics (e, “ring of six”). The brown trout Metaphase I meiosis shown in Figure 3B exhibits only long rod tetravalents involving 2 metacentrics and 2 acrocentrics, and no tetravalent rings. In these examples the brown trout has a chromosome number of 80 (20 metacentrics and 60 acrocentrics, common in brown trout populations, Zenzes and Voiculescu, 1975), while the rainbow trout has a chromosome number of 64 (40 metacentrics and 24 acrocentrics; interestingly among different populations there is a high degree of karyotype polymorphism related to Robertsonian fusion differences; Hartley and Horne 1982 and references therein, Thorgaard 1983). The prevalence of long rod tetravalents and lack of tetravalent rings is as expected given the lower number of metacentrics in brown trout versus rainbow trout.

Once one understands residual tetrasomy, it is easier to understand pseudolinkage that occurs in some salmonid families from males of mixed genetic background (see Figure 1). The essential difference is that the pairing of the telomeric homologous regions of the homeologous arms is not random but rather preferential for the homeologous arms from the same genetic background. We can use brook and lake trout as an example, where meiosis is being examined in male hybrids of these 2 species (splake trout). Arms cd, and g(gh) are from lake trout and arms ab, and e(ef) are from brook trout. The B locus is dropped for clarity as in Allendorf et al. (2015). The lake loci are A’_1_ and A’_2_ and brook loci are A_1_ and A_2_. The preferential homologous pairing of the telomeric regions of species-specific homeologous arms (note the dark green brook trout arms pairing and light green lake trout arms pairing), along with alternate disjunction will send arms cd (centromere 2) with e(ef) (centromere 3) to 1 meiotic pole and arms ab (centromere 1) with g(gh) (centromere 4) to the other pole. The result of this segregation will be non-parental gametes from this pseudolinkage (meiotic event), A_1_ (brook) with A’_2_ (lake) and A’_1_ (lake) with A_2_ (brook). Note: The 2 types of adjacent disjunctions would lead to only parental gametes on the one hand or unbalanced gametes on the other; neither of which have been observed. Again, the ratio of parental and non-parental gametes will be based on the ratio of bivalent pairing versus multivalent pairing. The excess of non-parentals observed in many studies is produced by pseudolinkage pairing occurring more often than bivalent pairing (or random tetravalent pairing).

In summary, we have shown and explained the simplicity and elegance of the Wright model to explain the chromosomal behavior behind residual tetrasomy in some male salmonid meioses, and how pseudolinkage is just a special case of residual tetrasomy. We have also shown that the Allendorf et al. (2015) model for residual tetrasomy involves all 4 arms in a pairing configuration that only provides 1 crossover between the telomeric regions of the homeologous arms versus the 2 crossovers in Wright’s model or the 2 crossovers in Allendorf et al.’s model for pseudolinkge. This model would not provide sufficient recombinants to get the segregation patterns they observe. Their models do not show a relationship between residual tetrasomy and pseudolinkage. Further, their pseudolinkage model, though technically correct for crossover locations, is meiotically unrealistic and misleading.

It is our hope that this detailed explanation will be useful to the many genomic studies of salmonid species (Brieuc et al. 2014; Berthelot et al. 2014; Gonen et al. 2014). The data gathered in detailed mapping studies should help us home in on where the zones of homology and homeology meet in particular homeologous arms and how these zones differ among the various species. We are now in the fortunate circumstance where the classical fields of cytogenetics and Mendelian genetics can combine with genomics and bioinformatics for new opportunities to understand chromosome evolution in the unique tetraploid derivative salmonid lineage.

## References

[CIT0001] Allendorf FW Bassham S Cresko WA Limborg MT Seeb LW Seeb JE 2015 Effects of crossovers between homeologs on inheritance and population genomics in polyploid-derived salmonid fishes. J Hered. 106:217–227. 25838153 10.1093/jhered/esv015

[CIT0002] Allendorf FW Danzmann RG 1997 Secondary tetrasomic segregation of MDH-B and preferential pairing of homeologues in rainbow trout. Genetics. 145:1083–1092. 9093860 10.1093/genetics/145.4.1083 PMC1207878

[CIT0003] Allendorf FW Thorgaard G 1984 Polyploidy and the evolution of salmonid fishes. In: Turner BJ, editor. The evolutionary genetics of fishes. New York (NY): Plenum Press; p. 1–53.

[CIT0004] Berthelot C Brunet F Chalopin D Juanchich A Bernard M Noël B Bento P Da Silva C Labadie K Alberti A 2014 The rainbow trout genome provides novel insights into evolution after whole-genome duplication in vertebrates. Nat Commun. 5:3657. 24755649 10.1038/ncomms4657 PMC4071752

[CIT0005] Brieuc MS Waters CD Seeb JE Naish KA 2014 A dense linkage map for Chinook salmon (Oncorhynchus tshawytscha) reveals variable chromosomal divergence after an ancestral whole genome duplication event. G3 (Bethesda). 4:447–460. 24381192 10.1534/g3.113.009316 PMC3962484

[CIT0006] Davisson MT Wright JE Atherton LM 1973 Cytogenetic analysis of pseudolinkage of ldh Loci in the teleost genus salvelinus. Genetics. 73:645–658. 17248602 10.1093/genetics/73.4.645 PMC1212921

[CIT0007] Gonen S Lowe NR Cezard2 T Gharbi K Bishop SC Houston RD Linkage maps of the Atlantic salmon (Salmo salar) genome derived from RAD sequencing. BMC Genomics 2014, 15:166. 24571138 10.1186/1471-2164-15-166 PMC4028894

[CIT0008] Hartley SE Horne MT 1982 Chromosome polymorphism in the rainbow trout (Salmo gairdneri Richardson). Chromosoma. 87:461–468. 7182125 10.1007/BF00333467

[CIT0009] Lee GM Wright JE Jr 1981 Mitotic and meiotic analyses of brook trout, *Salvelinus fontinalis* . J Hered. 72:321–327. 10.1093/oxfordjournals.jhered.a109354 6774006

[CIT0010] Lubieniecki KP Jones SL Davidson EA Park J Koop BF Walker S Davidson WS 2010 Comparative genomic analysis of Atlantic salmon, Salmo salar, from Europe and North America. BMC Genet. 11:105. 21092310 10.1186/1471-2156-11-105 PMC2995484

[CIT0011] May B Wright JE Stoneking M 1979 Joint segregation of biochemical loci in Salmonidae: Results from experiments with *Salvelinus* and review of the literature of other species. J Fish Res Board Can. 36:1114–1128.

[CIT0012] Morrison WJ 1970 Nonrandom segregation of two lactate dehydrogenase subunit loci in trout. Trans Am Fish Soc. 99:193–206.

[CIT0013] Thorgaard GH 1983 Chromosomal differences among rainbow trout populatons. Copeia. 1983:650–662.

[CIT0014] Wright JE Jr Johnson K Hollister A May B 1983 Meiotic models to explain classical linkage, pseudolinkage, and chromosome pairing in tetraploid derivative salmonid genomes. Isozymes Curr Top Biol Med Res. 10:239–260. 6354984

[CIT0015] Zenzes MT and Voiculescu I 1975 C-banding patterns in Salmo trutta, a species of tetraploid origin. Genetica. 45:531–536.

